# Unveiling the Latest Developments in Molecularly Imprinted Photocatalysts: A State-of-the-Art Review

**DOI:** 10.3390/polym15204152

**Published:** 2023-10-19

**Authors:** Adam Kubiak, Maria Stachowiak, Michał Cegłowski

**Affiliations:** Faculty of Chemistry, Adam Mickiewicz University, Uniwersytetu Poznanskiego 8, PL-61614 Poznan, Poland; adam.kubiak@amu.edu.pl (A.K.); marsta54@st.amu.edu.pl (M.S.)

**Keywords:** semiconductors, photocatalysis, molecularly imprinted, synthesis method, application

## Abstract

Responding to the growing concerns about environmental pollutants, scientists are increasingly turning to innovative solutions rooted in the field of environmental science. One such promising avenue combines the robustness of traditional photocatalysis with the precision of molecular imprinting, leading to the proposition of molecularly imprinted photocatalysts (MIPCs). These MIPCs hold the potential to specifically target and eliminate environmental pollutants, marking them as a promising tool in modern environmental remediation. As researchers delve deeper into this field, the design and optimization of MIPCs have become hotbeds for scientific inquiry. This comprehensive overview delves into the multifaceted approaches to MIPC design, elucidating on aspects like the selection of appropriate photocatalytic bases, the pivotal role of templates, the choice of monomeric building blocks, and the integration of effective cross-linking agents. However, as with all burgeoning technologies, the development of MIPCs is not without its challenges. These potential impediments to the successful innovation and implementation of MIPCs are also explored.

## 1. Introduction

The intertwined relationship between scientific innovations and the environmental and energy sectors has persisted for quite some time. Since the discovery of the Honda–Fujishima [1] effect back in 1972, researchers have been tirelessly working towards the development of efficient photocatalytic materials. These efforts have mainly focused on semiconducting materials as highly promising. The forefront of these efforts has been the utilization of TiO_2_ semiconductors, primarily due to their optimal band-gap width and favorable positions of valence and conduction bands [2,3]. Additionally, various original studies have been conducted to enhance photocatalytic performance, involving the synthesis of photocatalysts using nanomaterials such as ZnO, CdSe, g-C_3_N_4_, and others as the photocatalytic matrix [4,5,6].

However, an enduring problem that has persisted within this domain is the inherent lack of specificity exhibited by advanced oxidation processes (AOPs). This issue arises from the fundamental reliance of environmental pollutant (EP) degradation on hydroxyl radical reactions, which inherently lack discriminative characteristics and have elicited substantial international concerns [7,8]. In reaction to this challenge, researchers have diligently undertaken explorations into varied approaches aimed at enhancing both the discrimination and effectiveness of these processes. This has led to the emergence of molecular imprinted technology (MIT) [9,10]. This technique involves creating specific cavities in molecularly imprinted polymers (MIPs) using a template molecule. The outcome is a set of polymers endowed with the remarkable capability to selectively identify and adsorb specific molecules with high affinity. MIPs, often drawing parallels to natural molecular recognition systems like enzymes and substrates, or antigens and antibodies, offer distinct advantages [11,12,13]. These polymers are recognized for their robust stability, versatility, and ability to function effectively even in extreme environments. Such attributes have paved the way for the wide-ranging utilization of MIPs, spanning from solid-phase extraction to drug delivery. However, it is important to acknowledge that MIT is not the sole solution in the quest to address the limitations of AOPs. Several alternative strategies have been explored, and these strategies should not be overlooked. For instance, adsorption processes have gained attention as an effective means of removing contaminants from water sources, offering an alternative or complementary approach to photocatalysis [14,15]. Furthermore, researchers have been actively investigating the development of floating photocatalysts for wastewater remediation. These innovative materials are designed to maximize light exposure due to their extended surface area and are easily recoverable after use, making them highly sustainable and eco-friendly options. The utilization of floating supports derived from biosources adds an extra layer of environmental sustainability to this approach [16,17]. These developments reflect the diverse and evolving landscape of strategies for addressing environmental pollutant removal.

In light of these considerations, it is important to recognize that while MIT represents a promising approach to enhancing the specificity and efficiency of AOPs, it is not the sole path forward [18]. A comprehensive exploration of various strategies, including adsorption and floating photocatalysts, is essential for advancing the field of environmental remediation and ensuring sustainable and effective solutions for water pollution challenges.

When highlighting the benefits of molecularly imprinted photocatalysts (MIPCs), it is worth emphasizing that MIPCs exhibit an extraordinary level of specificity that is unparalleled in the field of photocatalysis. This specificity arises from the precision with which MIPCs are designed, creating molecular recognition sites within the photocatalytic matrix that are tailored to match the size, shape, and chemical properties of the target molecules of interest [19]. This exceptional selectivity empowers MIPCs to discriminate with remarkable accuracy, even in the presence of complex and diverse mixtures of contaminants. Such pinpoint targeting ensures that the photocatalytic action is focused on the substances that require remediation, mitigating the potential for unintended side reactions and minimizing energy consumption. Beyond selectivity, MIPCs are known for their exceptional stability and durability. These materials maintain their catalytic activity over extended periods, even under demanding environmental conditions [20]. This robustness makes them ideal candidates for continuous and long-term applications, reducing the need for frequent replacement and enhancing the overall efficiency and cost-effectiveness of remediation processes [21]. Additionally, MIPCs can be customized to possess outstanding adsorption capacity, enabling them to efficiently capture target molecules from aqueous solutions. This dual functionality as both a selective adsorbent and a photocatalyst is particularly advantageous in scenarios where rapid and effective removal of specific pollutants is crucial [22]. The synergy between selective adsorption and photocatalytic degradation ensures comprehensive and efficient pollutant removal. In alignment with the principles of sustainability and green chemistry, MIPCs can be fabricated using biosourced and renewable materials. Their synthesis processes can be optimized to reduce waste generation and energy consumption, contributing to environmentally responsible practices. Furthermore, the ease of regeneration and reusability of MIPCs reinforces their eco-friendliness, minimizing waste and resource consumption while maximizing their utility [23].

As the landscape of research continues to evolve, there has been a notable increase in studies centered around molecularly imprinted photocatalysts [24,25]. This trend is evident from the rising number of publications documented in the Web of Science (WOS). The past two years alone have witnessed a more than twofold surge in these publications. However, amidst this proliferation of research, the need for a comprehensive and unified understanding of the field remains essential for both researchers and professionals [26,27,28].

This review embarks on an exploratory journey into the realm of MIPCs. It delves into the fundamental principles of molecular imprinting and selective photocatalysis, navigating through the intricate pathways involved in designing and synthesizing MIPCs. Furthermore, the review sheds light on the diverse applications of selective photocatalysis, encompassing everything from eliminating organic compounds to creating cutting-edge photoelectrochemical sensors. This review’s ultimate goal is to bridge existing gaps and inspire further innovation, guiding the next phase of research and application in this dynamic domain.

## 2. Molecularly Imprinted Photocatalysts

Over recent years, the incorporation of semiconductor nanomaterials into the MIPs’ framework has become a subject of considerable interest. This endeavor is often accomplished through a diverse array of strategies, using several innovative techniques. These techniques can largely be categorized into three primary methods: surface imprinting [29], precipitation polymerization [30], and in situ polymerization [31].

Surface imprinting focuses on creating recognition sites on the surface of the semiconductors’ matrix, ensuring easy access and rapid binding of target molecules [32,33]. On the other hand, precipitation polymerization involves the formation of polymers in a dispersed phase by initiating polymerization in a homogeneous solution. This method often results in uniformly sized and shaped polymer particles, providing better consistency in the MIPs’ structure [34]. In situ polymerization is a technique where monomers are polymerized directly in the presence of the template molecule in the desired location, ensuring that the imprinted sites are well structured and precise [35].

In the subsequent sections, we delve deeper into these methods, emphasizing the pivotal insights and breakthroughs documented in scholarly articles. Through this detailed exploration, we aim to provide a comprehensive understanding of the intricacies involved in integrating nanomaterials with MIPs.

### 2.1. Surface Imprinting

**Figure 1:**
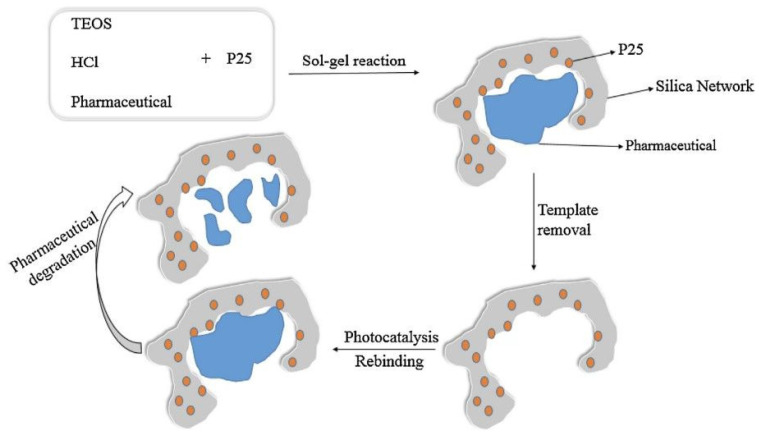
Preparation and evaluation of MI photocatalysts using the sol–gel technique. Reprinted with permission from ref. [37]. Copyright (2023), with permission from Elsevier (or applicable society copyright owner).

The prevalent technique for synthesizing these materials is the straightforward one-step sol–gel method, facilitating the production of photocatalysts in various forms, such as powders, fibers, or thin layers [36]. This method offers simplicity and adaptability for different templates. Its appeal lies in the ability to produce many molecularly imprinted materials without excessive solvent usage or complex multi-step procedures. The primary approach to template removal from imprinted regions is calcination, involving exposing the material to high temperatures (>300 °C) (Figure 1) [37]. Although concerns about potential distortion of imprinted sites or material degradation exist, calcination remains one of the most effective and frequently used methods [38]. An alternative approach is traditional extraction or ultrasound-assisted extraction, but these methods often necessitate high solvent consumption, which is undesirable.

**Figure 2:**
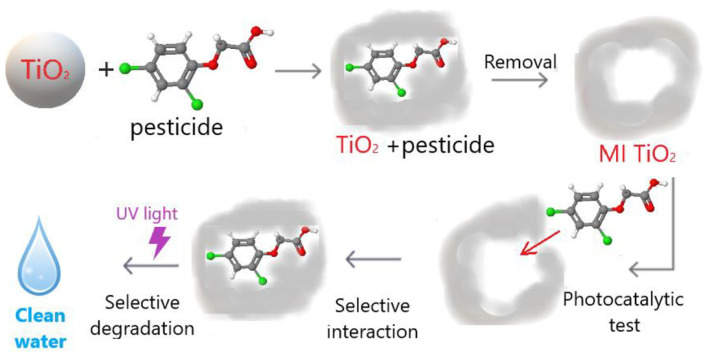
Schematic diagram of molecularly imprinted photocatalysts tailored for targeted elimination of pesticides. Reprinted with permission from ref. [36]. Copyright (2023), with permission from Elsevier (or applicable society copyright owner).

The growing industrial sector unfortunately contributes to increased soil and water pollution. Many hazardous toxins persist in the environment, posing challenges for natural elimination or requiring advanced purification systems. Faced with the pressing issue of water pollution, a significant portion of scientific research on photocatalysts focuses on their application, particularly in treating sewage to eliminate harmful and toxic pollutants. Consequently, the templates utilized in synthesizing molecularly imprinted catalysts primarily revolve around pesticides, pharmaceuticals, and their structural analogs [39,40,41], such as phenol derivatives like *o*-phenylphenol, a fungicide that, despite inhibiting fungal growth, poses health risks when present in wastewater [42]. The common agricultural pesticide 2,4-dichlorophenoxyacetic acid, known for its resistance to natural degradation and penetration into soil and water, is another frequently targeted compound [43,44]. Ethylparaben, a widely used preservative, also frequently appears in discussions due to its presence in water and sewage, which threatens human health. Besides toxins and pollutants, researchers sometimes employed structurally similar compounds as templates. For instance, Liu et al. [45] used 9-AnCOOH, a compound resembling potential pollutants, while Zhu et al. [46] used salicylic acid as a model impurity with an aromatic ring. Lee et al. [47] introduced an innovative approach, crafting a material comprising a quartz crystal coated with gold and an ultrathin layer of molecularly imprinted TiO_2_, showing promise in adsorbing 4-(4-propyloxyphenylazo)benzoic acid. This sparked further research into molecularly imprinted foils, created using the sol–gel method, for targeted substance detection and catalysis. An example of imprinted TiO_2_ development sans the use of organic polymers is demonstrated by Fiorenza et al. [42], which achieved the degradation of the pesticide 2,4-dichlorophenoxyacetic acid. The synthesis involved precipitating TiO_2_ from titanyl sulfate (TiOSO_4_) using HCl in the first step. Subsequently, a specific amount of template was added, and the mixture was stirred for an 1 h without external temperature control. The pH of the mixture was then adjusted to 1, and it was heated at 70 °C for a day. The resulting material, a white powder, underwent water washing and subsequent calcination at temperatures exceeding 300 °C to remove organic template residues. Fiorenza et al. [36] developed molecularly imprinted TiO_2_ for the degradation of the herbicide 2,4-dichlorophenoxyacetic acid (2,4-D) and the removal of the insecticide imidacloprid (1-(6-chloro-3-pyridinylmethyl)-nitro-2-imidazolidinimine) (Figure 2). The authors emphasized in their publication the simplicity and versatility of the sol–gel method for synthesizing molecularly imprinted materials. This approach involves combining all components in specific ratios and a proper sequence within a single vessel. The main constituents included the chosen template, acetic acid, ethanol, and titanium(IV) butoxide. The mixture was continuously stirred at room temperature until it reached a gel-like consistency, which was subsequently dried and calcined to eliminate the template residue. Fiorenza et al.’s extensive testing indicated the rejection of eventual toxicity in the synthesized titanium photocatalysts due to their exceptional performance.

**Figure 3:**
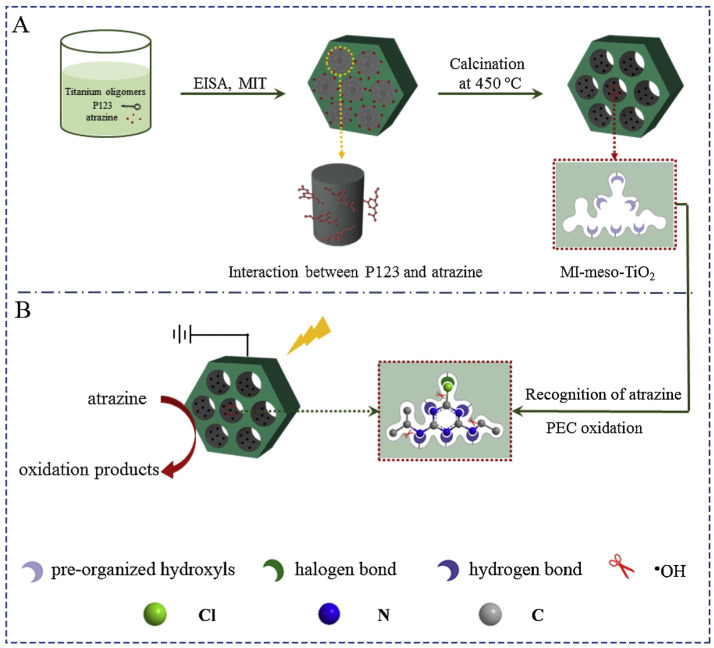
Fabrication process of MI-meso-TiO_2_ (**A**) and the enhanced and selective PEC catalytic behavior of MI-meso-TiO_2_ (**B**). Reprinted with permission from ref. [43]. Copyright (2023), with permission from Elsevier (or applicable society copyright owner).

Liu et al. [45] devised a method to prepare TiO_2_ nanotubes coated with a layer of molecularly imprinted titanium oxide, resulting in significantly enhanced photocatalytic activity and the ability to capture the target pollutant 9-AnCOOH on its surface. The researchers developed TiO_2_ nanotubes subjected to a surface sol–gel coating process. In this procedure, Ti(O-*n*Bu)_4_ was initially mixed with the appropriate amount of template molecules and solvents (toluene and ethanol). After 6 h of stirring, water was gradually added, and the mixture was stirred for an additional 6 h. Prepared nanotubes were introduced into the mixture under vacuum conditions. Following the addition of toluene wash and water hydrolysis, the coated nanotubes underwent purification from template residue through washing with ammonia aqueous solution and subsequent calcination at 500 °C. Notably, the obtained materials exhibited adjustable imprinted layer thickness by varying the number of dipping cycles in the reaction mixture, as highlighted by the promising feature of the results. In another investigation, Liu et al. [48] obtained imprinted TiO_2_ materials for the degradation of ethyl *p*-hydroxybenzoate (EP). Similar to other works, they utilized the classic sol–gel method by combining the TiO_2_ precursor with EP in the presence of an acid, allowing hydrolysis to form a gel. The reaction mixture was left in a dark environment for several days to facilitate gel formation and subsequently dried at 60 °C. This yielded yellow crystals that were powdered and subjected to calcination at 500 °C. The material exhibited enhanced effectiveness in removing EP from water. Shen et al. [49] formulated thin layers of molecularly imprinted TiO_2_ capable of degrading salicylic acid using the liquid-phase deposition (LPD) method. This technique involved immersing cleaned commercial borosilicate glass slides in a liquid mixture containing (NH_4_)TiF_6_, boric acid, HCl, salicylic acid, and P25 (an ingredient inhibiting crystallization). After thoroughly mixing the contents, the mixture was heated to 50 °C, and the prepared slides were placed in it for approximately 4 h. The final step involved removing the template molecules from the imprinted cavities, accomplished through extraction, photocatalytic oxidation, or calcination. Among these methods, calcination proved to be the most effective. This approach yielded a thin film of molecularly imprinted TiO_2_ on the glass slide’s surface, characterized by a satisfactory level of degradation of the target molecule. The method displayed increased selectivity with decreasing target impurity concentration, while the cleaning process had minimal impact on the structure of the resulting titanium surfaces. Zhu et al. employed salicylic acid as a template to develop a molecularly imprinted layer of TiO_2_ on glass slides’ surfaces [46]. Though similar to the previously described method, Zhu et al.’s procedure differed in the use of ammonium silicofluoride instead of P25 and a distinct polycondensation system temperature (35 °C for 18 h). To purify the final material from template residues, washing with water and acetone was conducted. Satisfactory results were obtained for both adsorption and catalytic activity, with confirmation provided by preparing a material imprinted with 2-hydroxyphenylacetic acid, which displayed a high affinity for its template molecule. Shi et al. [43] developed mesoporous molecularly imprinted TiO_2_, demonstrating effective oxidation efficiency for the herbicide atrazine (Figure 3). The approach involved obtaining molecularly imprinted titanium oxide and applying it to a purified Ti disc. The molecular imprinting process facilitated the accelerated separation of photogenerated charge carriers, leading to improved photocatalysis efficiency and increased adsorption capacity due to hydrogen and halogen bond formation. Similar work by Zhang et al. [44] involved plating a quartz crystal with a layer of molecularly imprinted TiO_2_ for the photocatalytic decomposition of atrazine, examining its degradation kinetics. The researchers further demonstrated that molecular imprinting onto TiO_2_ aids in the removal of specific enantiomers of chiral compounds, such as 2-(2,4-dichlorophenoxy)propionic acid (DCPP), which exists in both S and R forms [50]. The imprinted titanium layer was applied as a film on transparent conducting films (fluorine-doped tin oxide), utilizing a method akin to liquid-phase deposition. This approach showcased the versatility of molecular imprinting for various applications. A considerable number of molecularly imprinted photocatalysts employ silica as a matrix, producing a network with numerous cavities.

A study by de Escobar et al. [51] synthesized titanium photocatalysts coated with silica, designed to selectively attract rhodamine B (RhB) to their surface, which served as both the template and target molecule. The TiO_2_ precursor, TiCl_4_, was employed alongside the directly imprinted silica derived from TEOS. The researchers assessed the selectivity of the resulting materials towards rhodamine B (target), rhodamine 6G, and methylene blue. Their findings revealed that the molecularly imprinted silica domains exhibited a greater affinity for the target molecule compared to the commercial P25 material. Additionally, investigations were conducted to evaluate whether the analysis environment influenced material efficiency. Consequently, syntheses were performed concurrently, involving sol–gel syntheses under acidic, alkaline, and two-stage conditions, along with a non-hydrolytic environment involving SiCl_4_ and FeCl_3_ as a catalyst. The authors report that materials synthesized in acidic conditions exhibited a significant advantage in adsorbing rhodamine B, achieving an affinity coefficient even 180% higher than P25. The highest affinity coefficient and degradation level for the target molecule were achieved by the material synthesized using the acidic route and a commercial catalyst, leading to enhanced degradation. Further studies indicated that increasing template concentration during synthesis hindered removal and negatively affected catalytic efficiency. In alternate investigations, de Escobar et al. [52] crafted photocatalysts with low TiO_2_ content (7–16.60 mg/L) to degrade various pharmaceuticals: atorvastatin, diclofenac, ibuprofen, thioconazole, valsartan, ketoconazole, and gentamicin. Employing the imprinting method on silica and capitalizing on the complementary molecular cavities within the silica network and degraded substances, significant improvements in adsorption and degradation were noted. Moreover, these materials exhibited the potential for multiple reuses, as the authors employed them for up to seven cycles of photocatalysis, each cycle achieving an efficiency of around 60%. Ferreira et al. [53] developed core–shell microspheres featuring a titanium core enveloped in a molecularly imprinted silica layer. The procedure began with SiO_2_ microsphere preparation, involving a comprehensive mixture of TEOS, isopropanol, and aqueous ammonia solution, followed by drying. Subsequently, the microspheres were dispersed in isopropanol (or ethanol), and Igepal CA-630 was added. After thorough mixing for several hours, an alcohol suspension of titanium(IV) butoxide (TBOT) or titanium(IV) isopropoxide (TTIP) and bilirubin (template) was introduced and mixed again. Two synthetic routes were presented, one at room temperature and the other at 85 °C, both culminating in intensive washing of the imprinted TiO_2_ hollow microspheres. The resultant photocatalytic material demonstrated almost twice the rate of bilirubin degradation compared to the reference substance (protoporphyrin). Deng et al. [39] similarly produced a TiO_2_/SiO_2_ nanocomposite, with molecular imprinting aimed at 4-nitrophenol. The authors mixed tetrabutyl orthotitanate, tetraethyl orthosilicate, template, and acetic acid with ethanol. The mixture was cooled to 10 °C and subsequently raised to 140 °C, maintaining that temperature for 12 h. Template residue was removed using a Soxhlet extraction system. The process concluded with the lyophilization of the obtained material. Similar to other authors’ publications, the resulting photocatalyst exhibited a high potential for degrading the template impurity. Guo et al. [54] achieved a titanium material with imprinted silica via liquid-phase deposition for catalytic oxytetracycline (OTC) antibiotic degradation. The reaction mixture ((NH_4_)_2_TiF_6_, H_3_BO_3_, distilled water, oxytetracycline, with or without (NH_4_)_2_SiF_6_) was maintained at 38 °C for 20 h. A thin film formed on the vessel walls, which was then scraped, washed with water and ethanol, vacuum-dried, and calcined. The results of these studies demonstrated heightened photocatalytic activity of the materials and enhanced binding affinity for the target molecule.

As previously mentioned, an emerging trend in the exploration of molecularly imprinted photocatalysts involves their doping with other metal or non-metal compounds, aimed at imparting additional functional properties, such as paramagnetism or altering sensitivity to electromagnetic waves that stimulate pollutant degradation. Carboni et al. [38] obtained a mesoporous titanium-based film doped with La^3+^ ions that formed complexes with the template, bis-4-nitro-phenyl-phosphate, an organophosphate pesticide model. In one approach, a one-stage synthesis of dense molecularly imprinted La-doped titania films (dense-MIF) was executed by combining an alcoholic LaCl_3_·7H_2_O solution with a template and TiCl_4_ solution, immersing silicon substrates into the mixture, and subsequently heat-treating the formed coating layer. The second procedure introduced a mesoporous characteristic using the structuring agent Pluronic F127. Lanthanum’s robust interaction with the template increased the deposition of impurities near the catalyst, as affirmed by the authors’ research. Luo et al. [40] achieved a TiO_2_/WO_3_ nanocomposite molecularly imprinted for degrading 2-nitrophenol and a parallel material for 4-nitrophenol degradation. Employing a straightforward sol–gel synthesis and calcination, the researchers’ results validated the potential of the materials for water purification from harmful substances. Continuing research on the same templates, Wu et al. [41] employed the ethanol–water solvothermal method, vigorously mixing the template with ethanol, subsequently adding relevant components at room temperature, and then heating the mixture in an autoclave to 140 °C for 24 h. Wu et al. obtained nitrogen- and fluorine-doped molecularly imprinted titanium oxide, using NH_4_F as a doping agent. The researchers highlighted that molecular imprinting combined with doping accelerates charge separation and boosts adsorption, substantially amplifying the photocatalytic degradation efficiency. Moreover, shifting catalytic activity to visible light and employing inorganic matrices heightened stability and durability. Employing a similar double-doping technique, Zhu et al. [55] created a Ag/Zn/TiO_2_ composite material, showcasing the priority degradation of ethyl *p*-hydroxybenzoate. The addition of metal doping agents reduced imprinted titanium oxide particle size, thus improving catalytic performance. Molecular imprinting heightened the catalyst’s surface affinity, facilitating stronger hydrogen bonding with impurity molecules.

In recent years, the development of doped, molecularly imprinted photocatalysts has surged. One study saw Liu et al. [56] employ ethylparaben as a template to craft Ag-doped TiO_2_ molecularly imprinted photocatalysts, achieving a degradation rate of about 93% in 120 min. Other work led to a sulfur-doped titanium catalyst directly imprinted with active sites favoring ethylparaben, achieving 99% degradation of dyes—methyl orange and methylene blue—after 120 min [57]. Zhan et al. [58] obtained a molecularly imprinted titanium oxide modified with a Dawson-type nanocomposite, Co_3_P_2_W_18_O_62_·15H_2_O, demonstrating elevated photoactivity compared to reference TiO_2_ material. This enhancement was attributed to improved charge separation, electron-hole pair recombination inhibition, and increased active surface. Fiorenza et al. [59] investigated nitrogen-doped titanium photocatalysts imprinted with the *o*-phenylphenol fungicide, achieving satisfactory selectivity towards the template and catalytic performance enhancement in visible light. Zhang et al. [60] showcased a magnetic nanocomposite Fe_3_O_4_/Al_2_O_3_/molecularly imprinted TiO_2_, indicating elevated photocatalytic properties, controllable template adsorption, and magnetic properties for simplified photocatalyst separation from treated wastewater mixtures. Another approach to selective compound degradation involves doping and molecular imprinting on silica, yielding photocatalysts with complex synthesis procedures and multi-component structures. However, this approach remains less explored in the literature. Qi et al. [61] developed TiO_2_ mesocrystals doped with praseodymium, enhancing the photocatalyst’s ability to collect visible light and exhibit selective adsorption of the target pollutant. Similarly, Shen et al. [62] developed a TiO_2_ photocatalyst coated with molecularly imprinted silica and doped with aluminum oxide, demonstrating priority binding to target molecules and increased catalytic speed, even at low concentrations of the target pollutant diethyl phthalate (DEP). Han et al. [63] produced a TiO_2_-SiO_2_ composite catalyst doped with Cu^2+^ ions for 2,4-dichlorophenol degradation, utilizing the classic sol–gel method. While less common than SiO_2_, ZnO also serves as a classical catalyst for molecular imprinting. Cantarella et al. [64] focused on developing molecularly imprinted ZnO nanostructures using co-precipitation with ZnAc_2_·2H_2_O in the presence of the template molecule paracetamol. The results indicated complete degradation of paracetamol and substantial degradation of coexisting molecules. A study by de Escobar et al. [65] also delved into molecularly imprinted ZnO photocatalysts, adopting a modified synthesis approach to improve efficiency via doping with Al^3+^ ions. The results displayed high affinity for the template glycerol and efficient production of glyceraldehyde, highlighting the potential of molecularly imprinted photocatalysts for simultaneous purification and compound production.

In a comprehensive study on wastewater treatment, Xie et al. [66] developed a distinct molecularly imprinted NH_2_-MIL-53(Fe) (metal organic framework) catalyst tailored specifically for the targeted degradation of sulfamethoxazole within advanced oxidation processes. This catalyst stands out due to its ability to precisely identify and bind SMX, boasting a noteworthy removal rate of 38.04 mg/g. Such efficiency is largely due to weak interactions, especially hydrogen bonds, which pull SMX and its intermediary products to the catalyst’s surface, enhancing degradation. Furthermore, the researchers pinpointed ⋅OH as the main free radical involved in the degradation process. Interestingly, the imprinted layer of the catalyst did not influence the generation of this radical. This indicates that the catalyst’s design is centered on recognition without altering the catalytic process. These findings highlight the promising potential of incorporating MIP technology in AOPs, especially in tackling emerging micro-pollutants in wastewater, providing valuable perspectives for refining wastewater treatment techniques.

In recent years, the field of molecularly imprinted photocatalysts has witnessed significant progress, offering promising solutions to environmental and energy challenges. The straightforward sol–gel method remains a prevalent technique for MIPC synthesis, enabling the production of versatile photocatalysts in various forms. However, the distinction between standard semiconductor nanocomposites and imprinted counterparts poses a critical challenge, necessitating the development of advanced characterization techniques. Furthermore, the emerging trend of doping MIPCs with additional compounds enhances their functionality, although further research is needed for optimization. Additionally, MIPCs exhibit promise in AOPs for micro-pollutant removal in wastewater treatment. As the field continues to evolve, addressing these challenges will be crucial to advancing the effectiveness and applicability of MIPCs in environmental remediation and energy-related applications.

A summary of recent developments in surface imprinting is presented in Table 1.

### 2.2. Molecularly Imprinted Polymers on Photocatalysts

Over the past ten years, researchers have shifted their attention to improving surfaces by layering a slender film of molecularly imprinted polymers (MIPs) on semiconductors, notably titanium dioxide [68].

In essence, molecular imprinting is a technique for creating predefined recognition cavities in synthetic polymers that have inherent selectivity for specific template molecules. During the creation process, functional building blocks surround the template, establishing connections through their functional groups [69]. Post initiation, this complex intertwines with a cross-linking agent, resulting in the formation of an imprinted structure. These interconnected materials are identified as MIPs [70]. Once the template molecule is extracted under specific conditions, cavities that mirror the size and configuration of the original template molecules emerge [71]. In scientific literature, various templates such as medications, contaminants, food components, peptides, proteins, and other derivatives have been employed for molecular imprinting. MIPs crafted with contaminants as the templates are especially captivating in environmental chemistry [72,73]. They offer the prospect of designing advanced materials adept at selectively removing highly toxic organic pollutants in diluted settings, even amidst prevalent pollutants in elevated concentrations. Achieving this necessitates the crafting of water-friendly MIPs. Zhang [74] provided a concise overview of methodologies to generate MIPs: (1) optimizing conditions within the conventional molecular imprinting method; (2) incorporating specific polymerizable monomers; (3) employing surface post-modification techniques; (4) resorting to Pickering emulsion polymerization; and (5) applying controlled/living radical precipitation polymerization [37]. Of these, the Pickering emulsion polymerization stands out, given water molecules (integral to the Pickering emulsion polymerization’s continuous phase) actively partake in the imprinting phase [75,76]. Beyond water-adaptable MIPs, current advancements in physically encasing and chemically affixing MIP particles, specifically for clean water applications, alongside challenges in deciphering molecular imprinting mechanisms, have been explored [77,78]. Such research endeavors foster the utilization of MIPs in selectively extracting toxic organic pollutants from intricate wastewater matrices.

Liu et al. [79] introduced a novel approach in photocatalysis by synthesizing heterojunction magnetically imprinted PEDOT/CdS photocatalytic nanoreactors using a microwave-assisted surface imprinting technique. Emphasizing the pivotal role of molecular imprinting, this method showcased the PEDOT printed layer’s capability for three-dimensional (3D) specific recognition and selective photocatalysis of danofloxacin mesylate. The nanoreactor achieved an impressive specific selectivity coefficient and enhanced photocatalytic activity with a degradation rate of around 84.84%. One of the standout attributes was the PEDOT layer’s ability to reduce the photocorrosion and secondary contamination of CdS. Furthermore, the PEDOT/CdS heterostructures demonstrated dual proficiency in recognizing specific pollutants and mitigating CdS photocorrosion in water environments. This innovative approach, combining magnetic imprinting with enhanced photocatalytic performance, signifies a major advancement in pollutant degradation.

**Figure 4:**
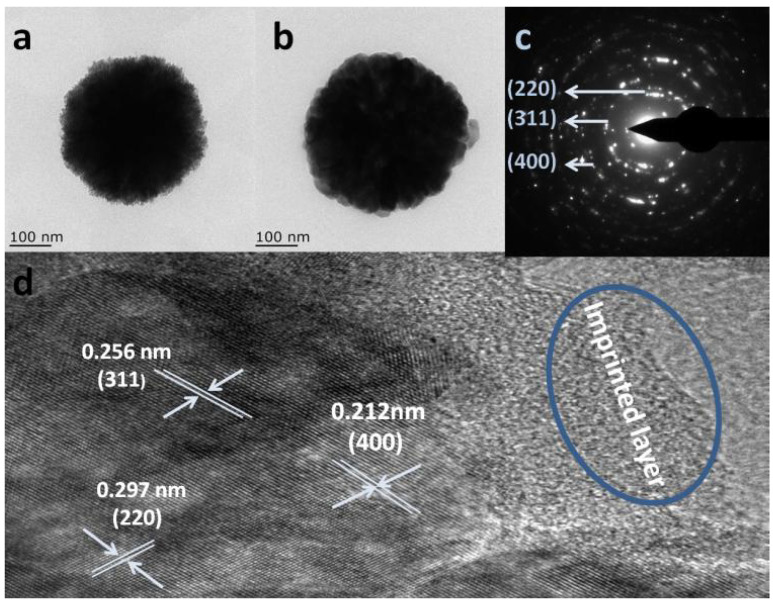
TEM images of CoFe_2_O_4_ (**a**) and the magnetic ion-imprinted heterojunction photocatalyst (**b**), with the SAED pattern (**c**) and a high-resolution TEM snapshot (**d**) of the magnetic ion-imprinted heterojunction photocatalyst. Reprinted with permission from ref. [80]. Copyright (2023), with permission from Elsevier (or applicable society copyright owner).

Using the microwave-assisted ion imprinting method, He et al. [80] successfully synthesized a magnetic ion-imprinted heterojunction photocatalyst (Figure 4). The procedure encompassed the dispersion of carboxylated CoFe_2_O_4_ in dimethyl sulfoxide, followed by the integration of polyo-phenylenediamine. They then introduced Cu(NO_3_)_2_·3H_2_O, EGDMA, AIBN, and P123. This combination was exposed to a microwave reaction with set parameters (600 W, 70 °C, 90 min) to yield the targeted photocatalyst. A distinctive feature of this product are its twin functionalities: the ability to selectively reduce Cu^2+^ ions and concurrently degrade tetracycline. This dual functionality is attributed to the Cu^2+^ imprinted cavities in its layer and a mesoporous layer within. A significant observation was its pronounced selectivity towards Cu^2+^ ions over other competitors, underscoring its superior specificity. The collaborative effect of CoFe_2_O_4_ and POPD in forming a heterojunction structure was crucial in facilitating the effective separation of electrons and holes, amplifying its photocatalytic prowess.

Huang et al. [81] unveiled magnetically recoverable adsorbent, molecularly imprinted magnetic γ-Fe_2_O_3_/crosslinked chitosan composites (MIPs), crafted using the microemulsion method. The formulation of MIPs is complex, entailing a fusion of chitosan and γ-Fe_2_O_3_ with a NOR solution, subsequently dissolved in acetic acid. This concoction is merged into a paraffin base with an emulsifier, sequentially treated with methanal and glutaraldehyde, pH-balanced, and subjected to several washes before oven-drying. The analogous non-imprinted polymers (NIPs) are crafted in a comparable manner, but without the template. The study primarily delved into the adsorption and Fenton-like oxidative degradation of a pharmaceutical impurity, norfloxacin (NOR), utilizing MIPs. Notably, MIPs demonstrated enhanced specificity in NOR adsorption over the conventional antibiotic sulfadiazine. Moreover, when initiating a Fenton-like reaction, the pairing of MIPs with H_2_O_2_ dramatically accelerated the NOR elimination compared to γ-Fe_2_O_3_/H_2_O_2_. A remarkable attribute of these MIPs is their magnetic reusability after the process. This research highlights the promising avenue of integrating Fenton reactions with magnetic MIPs for pharmaceutical waste treatment.

Chi et al. [82] synthesized a tailored metal-organic framework integrated with an imprinted layer, referred to as Fe(II)-MOFs@MIP. This cutting-edge structure was obtained using dibutyl phthalate (DBP) as a representative substance, demonstrating its outstanding proficiency in targeted adsorption and breakdown. The creation of Fe(II)-MOFs@MIP involved ultrasonically mixing 1.0 g of Fe(II)-MOFs with acetonitrile, resulting in Solution A. Simultaneously, DBP and MAA were sonicated in acetonitrile, generating Solution B. These solutions were merged, and EGDMA was added, followed by refrigeration. The next step introduced AIBN and was complemented by a nitrogen influx to expel oxygen. A sustained 12 h heat treatment at 70 °C was executed. Post heating, the compound was filtered, subjected to a methanol wash, and meticulously cleaned with a blend of methanol and acetic acid. The refined Fe(II)-MOFs@MIP underwent an ethanol rinse to rid any remaining acetic acid before drying. UV-vis examinations indicated that hydrogen bonding and π-π interactions were crucial for DBP recognition. Additionally, in situ Raman analyses, along with radical confinement studies, highlighted the imprinted layer’s efficiency in effectively sequestering pollutants. This strategic confinement significantly shortens the journey for free radicals, positioning Fe(II)-MOFs@MIP as a hopeful contender against stubborn, low-concentration organic contaminants.

In a study by Wang et al. [83], TiO_2_ films were crafted utilizing acetaminophen as a guiding molecule and p-tert-butyl calixarene as a key monomer, synthesized in a mixture containing (NH_4_)_2_TiF_6_ and H_3_BO_3_. Notably, a simple ethanol rinse was sufficient to eliminate the acetaminophen imprint. In another research, Wang et al. [19] produced tetracycline hydrochloride (TC) molecular imprints on modified TiO_2_ nanotubes. This was achieved by introducing the TC molecule to a precursor mix of (NH_4_)_2_TiF_6_ and H_3_BO_3_. The resulting structures displayed enhanced affinity for the original template molecules. Feng’s group [60] chose L-glutamic acid (GA) for their study, crafting GA-patterned TiO_2_ films through a liquid-phase technique, employing (NH_4_)_2_TiF_6_. Separately, Tatemichi et al. [84] utilized pepsin as a blueprint molecule. This led to the deposition of nano-TiO_2_ alongside a composite formed of pepsin and polylysine on a gold base. After this procedure, they extracted pepsin, leaving a nano-layer with specific pepsin-shaped cavities. In research by Xu et al. [12], a special TiO_2_ imprint combined with magnetic Fe_3_O_4_ nanoparticles was synthesized. This utilized the LPD approach with estrone as the guiding molecule. Interestingly, post-synthesis exposure to UV light enabled the easy removal of the estrone imprint. Shen et al. have created photocatalysts through an in situ polymerization of oxylipin 12-oxo-phytodienoic acid (OPDA) in conjunction with target molecules and TiO_2_ nanoparticles. This method stands out from conventional MIP preparations as polymerization is activated photocatalytically using UV light on TiO_2_ nanoparticles, bypassing the need for additional chemical initiators. Notably, they have developed a technique where UV light facilitates the removal of the template from the polymer layer, yielding MIP-coated TiO_2_ as the end product. TEM studies showcase the MIP layer’s thickness at about 5 nm.

The realm of MIPC synthesis encompasses a diverse array of methods, each possessing its unique characteristics and applicability. One such method is emulsion polymerization, which requires the use of appropriate emulsifiers to ensure the formation of fine emulsion droplets [85]. Subsequently, the introduction of initiators triggers cross-linking polymerization, resulting in the production of spherical polymers with uniform particle sizes. Another notable approach for preparing molecularly imprinted photocatalysts involves in situ polymerization. To illustrate the practical application of MIPs in photocatalysis, let us consider the example of photocatalytic nitrogen fixation [86]. In this study, non-metallic boron and noble metal silver-modified graphitic carbon nitride were synthesized using a direct in situ decomposition-thermal polymerization method. This innovative technique utilized dicyandiamide, boric acid, and silver nitrate as precursor materials, leading to the formation of Ag/B-doped g-C_3_N_4_ composites [87]. These composites, particularly those with an optimal mass ratio, demonstrated a remarkable enhancement in photocatalytic ammonia production under simulated sunlight illumination. The improved performance was attributed to several factors, including a reduced band gap, the development of mesopores resulting from boron doping, and the presence of loaded silver nanoparticles. Notably, the latter contributed to the inhibition of photogenerated charge carrier recombination via a heterojunction interface. Furthermore, Yang et al. [88], in their research, designed and fabricated a donor-acceptor conjugated polymer/TiO_2_ heterojunction catalyst with strong interfacial interactions using a facile in situ thermal treatment. To expand the light-harvesting window, soluble conjugated copolymers with D-A architecture were prepared by Pd-mediated polycondensation of diketopyrrolopyrrole (DPP) and t-butoxycarbonyl (t-Boc) modified carbazole (Car). These copolymers served as visible-light-harvesting antennas, effectively coupling with TiO_2_ nanocrystals.

The field of molecularly imprinted photocatalysts is witnessing significant progress in its quest to address environmental and energy challenges. Researchers have explored various synthesis methods and practical applications, demonstrating the versatility and potential of MIPCs in these domains. Notable advances include the development of MIPCs for targeted adsorption and degradation of specific pollutants, as well as innovations in enhancing photocatalytic performance using techniques like in situ polymerization and heterojunction catalyst design.

However, one critical issue in the field of MIPCs is the limited differentiation between standard semiconductor nanocomposites and their imprinted counterparts. Current characterization techniques, while valuable, may not provide sufficient distinction, necessitating the development of real-time or continuous monitoring methods to understand the unique behaviors of imprinted photocatalysts. Moreover, ensuring strong and specific interactions between templates and monomers is essential for the success of MIPCs. Advanced synthesis methods, such as controlled radical polymerizations, are needed to optimize the affinity and binding capabilities of these materials.

These critical issues underscore the need for ongoing research and innovation in the field of MIPCs to advance their effectiveness in addressing environmental pollution and energy-related challenges.

#### Monomers and Cross-Linkers

Building upon traditional methodologies employed in MIPC creation, a diverse selection of monomers and cross-linkers—namely, acrylic acid, methacrylic acid, acrylamide, trimethylolpropane trimethylacrylate, and ethylene glycol dimethacrylate—have become the preferred choices for the formation of MIPs [89,90]. When integrated, these elements often create a protective and inert layer over semiconductor surfaces. Among these, methacrylic acid displays a moderate affinity to water. Yet, compounds such as poly(EGDMA-MAA) are noted for their hydrophobic tendencies, which can influence and sometimes hinder their ability to degrade selectively in aquatic environments [81,90].

Recent explorations, as documented by researchers including Li et al. [91] and Zhu et al. [92], have shed light on the capabilities of MIPCs constructed using ingredients like acrylamide. Their findings highlight not only the remarkable absorption rates but also the impressive selectivity these configurations offer. Delving into the realm of hydrophilic elements, scholars like Bao et al. [93] and Huang et al. [94] have been pioneering in their approach. They have been investigating the potential of water-attracting monomers and cross-linkers for the tailored design of MIPCs. Their end goal is a focused approach towards the selective extraction of EPs from multifaceted wastewater systems. Based on these research insights, it appears that foundational monomers like acrylamide and *N*-isopropyl acrylamide may outperform methacrylic acid in terms of their suitability for crafting MIPCs. Further, the strategic introduction of water-friendly cross-linkers could play a pivotal role in enhancing the water adaptability of MIPCs. Pushing the boundaries of innovation, studies by Luo et al. [95] underscore the promising research of incorporating electrically conductive monomers. This approach, aimed at designing a composite heterojunction structure with semiconductors, has yielded interesting results. For instance, photocatalysts like TiO_2_/CNDs/MIP, developed using the versatile OPDA through advanced surface molecular imprinting techniques, stand out for their robust binding capacity. In a similar vein, novel compounds such as poly-3,4-ethylenedioxythiophene are making waves in the field, primarily for their role in crafting molecularly imprinted heterojunction photocatalysts that are adept at utilizing visible light [96]. Looking ahead, given the intrinsic ability of these compounds to serve as efficient electron donors in visible light conditions, there is a growing consensus in the scientific community that conductive polymers and related derivatives might play a more central role in future MIPCs designs. However, despite these advancements, the intricate mechanisms underpinning their photocatalytic actions remain somewhat enigmatic, underscoring an urgent need for comprehensive and nuanced research in this burgeoning domain.

In summary, recent research has explored a range of monomers and cross-linkers for molecularly imprinted photocatalyst (MIPC) creation, emphasizing the need to strike a balance between affinity and structural integrity in MIPs. Notably, acrylamide and water-attracting monomers show promise for improving selectivity and water adaptability. Additionally, the incorporation of electrically conductive monomers has opened new avenues for enhanced binding capacity and visible light utilization in MIPCs. However, the precise mechanisms governing these photocatalytic actions require further investigation. These critical points underscore the ongoing challenges and opportunities in advancing MIPC technology for environmental pollutant removal.

A summary of recent developments in molecularly imprinted polymers on photocatalysts is presented in Table 2.

## 3. Applications

Over recent decades, the advancement of selective photocatalysis has led to the creation and application of various MIPCs to address environmental challenges. These MIPCs, under specific light conditions, exhibit a selective attraction to target molecules, facilitating their removal through oxidation or reduction processes. Additionally, some MIPCs, with their electrochemical-response capabilities and distinct recognition properties, have been the foundation for the development of a number of photoelectrochemical sensors using imprinted semiconductors.

### 3.1. Photo-Oxidation of Toxic Organic Pollutants

**Figure 5:**
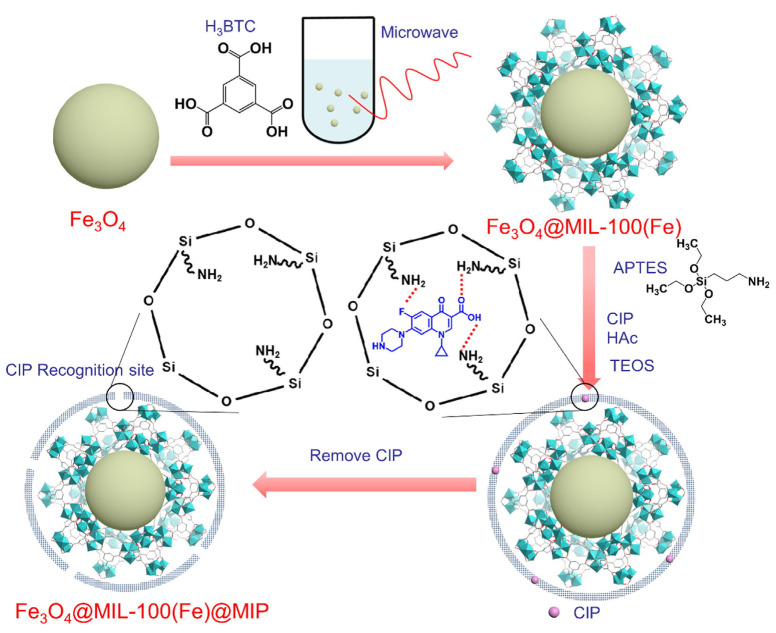
Diagram illustrating the synthesis process of MOF-centered core–shell porous molecularly imprinted nanospheres. Reprinted with permission from ref. [78]. Copyright (2023), with permission from Elsevier (or applicable society copyright owner).

Pharmaceutical contaminants present serious threats to aquatic ecosystems and human well-being. One pressing challenge is the targeted collection and breakdown of these pollutants in genuine wastewater systems. In response, Li et al. [78] used mesoporous carriers based on MIL-100, combined with surface molecularly imprinted polymers. This innovative approach showcased unmatched selectivity and an unprecedented uptake capacity for ciprofloxacin (Figure 5). Notably, this advanced material efficiently extracted the specified compound from real Beijing wastewater. Moreover, its iron-centric active sites enabled the full disintegration of the compound under visible light with just a trace of hydrogen peroxide.

He et al. [97] employed rhodamine B (RhB), creating surface molecularly imprinted polymers (MIPs) named MIPsRhB–PPy/TiO_2_. Experimental results demonstrated that this composite displayed improved affinity, absorption capacity, and swift absorption rates specifically for RhB, highlighting its remarkable recognition selectivity. Particularly noteworthy, the rate constant for the photodegradation of RhB on MIPsRhB–PPy/TiO_2_ was measured at 0.0158 min^−1^, a notable 3.6 times higher than that of NIP-PPy/TiO_2_. This illustrated the heightened precision of RhB photocatalysis under visible light conditions. In a study by Liu et al. [98], the RhB template was utilized to synthesize MIPs/Co-TiO_2_ nanocomposites. These nanocomposites exhibited an outstanding rate constant value of 0.03606 min^−1^ for RhB photodegradation, surpassing the performance of both Co–TiO_2_ nanoparticles and NIP/Co–TiO_2_ alternatives.

In research by Xu et al. [12], molecularly imprinted TiO_2_, combined with magnetic Fe_3_O_4_ nanoparticles, was crafted using estrone as the guiding template through the LPD technique. Highlighting its ability to selectively degrade estrone under UV light exposure, the resultant Fe_3_O_4_@SiO_2_@imprinted TiO_2_ displayed notable adsorption selectivity, rapid kinetics, and sustained stability in prolonged photocatalysis. Its peak adsorption of estrone stood at 2.62 mg/g, spotlighting its potential for effectively tackling minuscule organic pollutants amidst dominant pollutants. Zhang et al. [99] developed imprinted polymer-modified TiO_2_ nanotubes (S-MIP-TiO_2_ NTs) utilizing 17-β-estradiol as a template, emphasizing its adsorption capacity from 10 ng/L to 1000 mg/L. These nanotubes exhibited a stronger adsorptive nature and specificity for the photocatalytic breakdown of 17-β-estradiol at sparse concentrations compared to unmodified TiO_2_, making them potent tools in handling estrogen compounds in urban wastewater. 

Diclofenac (DCF), a commonly used non-steroidal anti-inflammatory drug (NSAID), has become a matter of global concern due to its widespread presence in water sources. Researchers have consistently detected DCF in the effluents of wastewater treatment plants and surface water, even at concentrations as low as several μg/L or ng/L, which underscores the urgency of addressing its potential adverse effects on human health and ecosystems [107]. Moreover, DCF’s high log K_OW_ value, around 4.5, means it can bioaccumulate in the tissues of organisms, further amplifying its toxicity. Given these environmental and health concerns, it has become crucial to develop innovative technologies for the removal of DCF from water sources. Among the various methods explored, advanced oxidation processes (AOPs) have gained recognition as highly effective tools for eliminating organic pollutants, including DCF. Within the realm of AOPs, visible-light-driven (VLD) photocatalytic oxidation has shown promise due to its exceptional efficiency and potential utilization of solar energy. Several types of photocatalysts have been developed for DCF removal, including TiO_2_-based [108], Bi-based [109], and Ag-based semiconductors [110], all of which have demonstrated efficiency in breaking down DCF molecules under the influence of light. However, a concern with many of these photocatalysts is the potential release of heavy or noble metal ions, which could lead to secondary pollution if these metals are released into the environment [111]. Consequently, there is a growing interest in developing metal-free photocatalysts, often referred to as “green” materials, to facilitate large-scale applications while minimizing environmental risks. In response to these challenges, a recent study led by de Escobar et al. [100] introduced an innovative approach to address DCF contamination in water sources. The researchers designed a novel photocatalyst by combining TiO_2_ with Cu_2_O-doped TiO_2_, specifically tailored for DCF degradation. This novel approach incorporated imprinted polymers, which enhanced the selectivity and efficiency of the photocatalyst for DCF degradation. When exposed to UV light, the imprinted variant exhibited significantly higher degradation rates compared to the non-imprinted one, indicating a promising solution to mitigate DCF pollution in water systems. This development represents a significant step forward in the quest to tackle the environmental impact of DCF and underscores the importance of continuous research and innovation in water treatment technologies.

Norfloxacin, an antibacterial agent, is extensively used worldwide, leading to environmental residues. Research by Li et al. [112] employed a molecular imprinting method on TiO_2_ particles, yielding impressive results. The imprinted polymers showed greater affinity and selectivity for norfloxacin and related compounds than conventional TiO_2_. Additionally, these MIPs maintained their efficacy over multiple reuse cycles, highlighting their potential for water purification against antibacterial residues.

Perfluorinated chemicals (PFCs) are recognized for their robust chemical stability and the potential to persist and bioaccumulate, posing threats to human and aquatic life [113]. Notably, compounds like perfluorooctanoic acid and perfluorooctanesulfonate are found in various water bodies and wastewater treatment plants. In a study by Wu et al. [27], developed TiO_2_ nanotubes embedded with molecular imprints using specific agents and PFOA as the primary model. Their tests revealed the MIP-TiO_2_NTs demonstrated enhanced PFOA degradation and specificity compared to their counterparts. This offers a promising approach to address fluorinated chemical pollutants.

Wu et al. [41] developed an N-F co-doped MIP-TiO_2_, referred to as MIP-NFTs, using an ethanol-hydrothermal approach. This demonstrated superior photocatalytic efficacy under artificial sunlight, especially for pollutants like 2-NP and 4-NP, when contrasted with non-imprinted forms. Specifically, the 2-NP and 4-NP adsorption rates on the respective MIP-NFTs were approximately 1.78 and 2.21 times greater than on the NIP-NFTs. Selectivity tests for degradation revealed coefficients of 1.93 for 2-NP relative to 4-NP and 1.61 for 4-NP relative to 2-NP on their designated MIP-NFTs. Rate constants for the photodegradation of 2-NP and 4-NP on their matching MIP-NFTs were recorded at 0.05233 min^−1^ and 0.03734 min^−1^, respectively, indicating enhancements of 267% and 198% when compared to NIP-NFTs under simulated sunlight conditions.

Understanding the mechanism of photo-oxidation is crucial for environmental remediation. This process hinges on photons interacting with a specialized material known as a photocatalyst, a pivotal component in MIPCs [114]. When these photons possess sufficient energy, they initiate the formation of electron–hole pairs, initiating a sequence of reactions on the photocatalyst’s surface. These electron–hole pairs are highly reactive and assume a vital role in the breakdown of noxious organic pollutants. Among the cast of reactive characters, reactive oxygen species (ROS) such as hydroxyl radicals (^•^OH) and superoxide radicals (^•^O^2−^) shine brightest, renowned for their potent capacity to oxidize organic contaminants aggressively [115,116].

MIPCs introduce a new dimension to the photocatalytic mechanism. MIPSs function as highly selective filters meticulously tailored to match the size, shape, and properties of specific pollutants [117]. They collaborate with the photocatalyst, initially capturing target pollutants from the surrounding environment. This capture sets the stage for the photo-oxidation process. By positioning the pollutants in close proximity to the ROS generated on the photocatalyst’s surface, MIPs enhance the likelihood of direct oxidative interactions [118,119]. This, in turn, expedites the degradation of pollutants into simpler, less harmful substances. Through selective adsorption and photo-oxidation, MIPCs zero in on particular substances, reducing side reactions and conserving energy. This comprehension serves as the cornerstone for pioneering solutions to environmental pollution challenges, rendering the cleanup process more efficient and sustainable.

### 3.2. Electrochemical Sensors

Examining the transformation of light energy into chemical and electrical energies, photochemistry (PEC) sensors emerge as prominent tools. These instruments operate when photoactive materials engage with analytes in the presence of light. The relationship between the photocurrent or photovoltaic voltage and the concentration of the analyte enables accurate measurement [55,120]. Consequently, titanium dioxide is a favored photoactive metal oxide, and its combination with MIPs enhances its photoelectrical performance [121,122].

**Figure 6:**
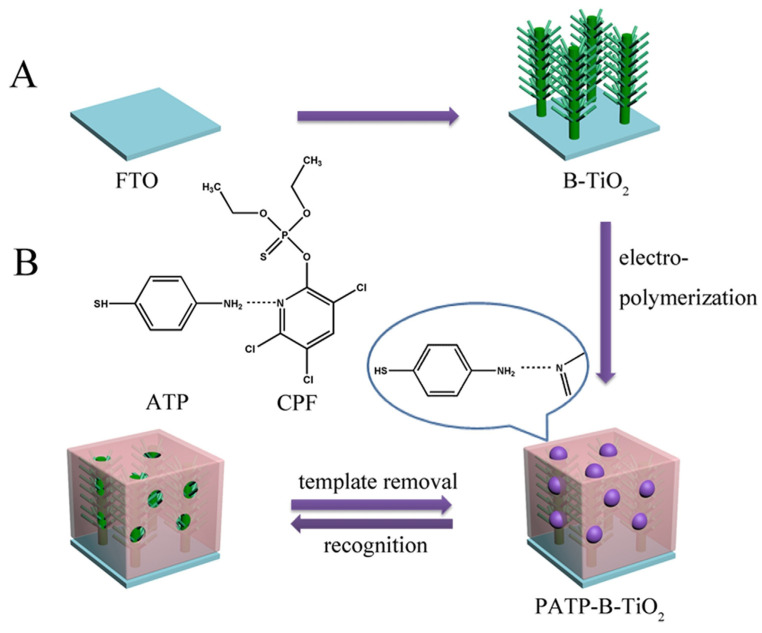
(**A**) The fabrication process of the PEC sensor. (**B**) Schematic illustration for the hydrogen bond interactions between ATP and CPF. Reprinted with permission from ref. [103]. Copyright (2023), with permission from Elsevier (or applicable society copyright owner).

Thanhthuy et al. [77] created a distinctive PEC sensor by incorporating a selective layer onto meticulously arranged nanotube arrays. The sensor displayed remarkable sensitivity and selectivity towards PFOS in water, undeterred by other higher concentration pollutants. This innovation reaffirms the potential of PEC sensors in environmental monitoring. Shi et al. [101] formulated a PEC sensor using modified TiO_2_ nanotubes to discern 2,4-D, a potential endocrine disruptor, demonstrating a noteworthy detection limit. A similar endeavor by Lu’s team yielded a PEC sensor adept at detecting another endocrine disruptor, BPA, emphasizing the potential of such sensors in monitoring environmental disruptors. Wang et al. [102] adopted a unique approach by imprinting *o*-phenylenediamine monomers and chlorpyrifos on gold nanoparticle-enhanced TiO_2_ nanotubes for PEC sensing. This sensor exhibited high specificity and potential for monitoring organochlorine pesticides in green produce. In another venture, researchers constructed a PEC sensor targeting lindane, an organochlorine pesticide, by utilizing aminothiophenol. This MIP-based PEC sensor showcased remarkable specificity and is a promising tool for detecting lindane in practical samples. Liu et al. [67] developed an advanced photoelectrochemical (PEC) sensor using MC-LR as a model molecule, integrating molecularly imprinted TiO_2_ with multi-walled carbon nanotubes (MI-TiO_2_@CNTs) using the sol–gel process. This MI-TiO_2_@CNT PEC sensor, capable of detecting from 1.0 pM to 3.0 nM with a threshold of 0.4 pM, showed superior photo-oxidation efficiency for MC-LR compared to traditional TiO_2_ and non-imprinted variants. The sensor’s heightened photocurrent response and selectivity make it ideal for future PEC applications. The success of this MC-LR sensor is attributed to the combination of the unique molecular imprinting on its surface and the excellent photoelectric characteristics of the TiO_2_@CNTs heterojunction structure. Sun et al. [103] introduced a PEC sensor based on a molecularly imprinted polymer (MIP) combined with hierarchical branched TiO_2_ nanorods (B-TiO_2_ NRs), specifically designed for sensitive CPF detection (Figure 6). Produced via a hydrothermal method, this sensor can detect CPF concentrations ranging from 0.01 ng·mL^−1^ to 100 ng·mL^−1^ with a minimum detection limit of 7.4 pg·mL^−1^. The core components, B-TiO_2_ NRs, were anchored with an MIP, which was confirmed through electron microscopy and voltammetry. The sensor’s effectiveness is due to the inverse relationship between its photocurrent response and CPF concentration. Moreover, its specialized design ensures superior CPF recognition, even amidst other pesticides, showcasing its potential for detecting environmental toxins with high sensitivity, specificity, and stability.

### 3.3. Other Applications

Geng et al. [104] created a nano-TiO_2_-based molecular imprinting polymer using propazine and several other agents. This MIP effectively recognized and adsorbed promethazine, and showed notable cross-reactivity with chemicals like simazine and atrazine. These MIPs, when used as extraction materials, allowed the precise isolation and measurement of pesticide residues in various samples using high-performance liquid chromatography. This approach aids in the precise detection of trace residues in different environments and food items.

Khoddami et al. [105] introduced a magnetic molecularly imprinted polymer using 3-(2-aminoethylamino) propyltrimethoxysilane and other ingredients to target Co(II) ions. This compound was found to be both stable and reusable, as its adsorption efficiency remained consistent after multiple tests. The preparation was straightforward, and the performance aligned well with established kinetic and adsorption models. The compound’s selectivity and performance make it a potential tool for determining Co(II) levels in various samples.

In research carried out by Zhao et al. [106], they successfully developed lysozyme (Lyz)-imprinted TiO_2_ nanoparticles, referred to as TiO_2_@Lyz-MIPs, making use of ionic liquids, specifically choline dihydrogen phosphate. This substance played a crucial role in stabilizing Lyz throughout the process. The creation was made possible using a free radical polymerization method that utilized multiple components. A significant finding was that when TiO_2_ was modified using polydopamine (PDA) and choline dihydrogen phosphatewas applied, there was a marked improvement in the nanoparticles’ Lyz adsorption capabilities. The best conditions resulted in an imprinting value of 4.40 and a peak adsorption limit of 120 mg g^−1^ for the TiO_2_@Lyz-MIPs, which was considerably higher than those of non-imprinted alternatives. Additionally, TiO_2_@Lyz-MIPs showcased their ability to efficiently isolate Lyz from watered-down egg white. Such results point to the development of distinct recognition sites on the surface of the TiO_2_ nanoparticles, emphasizing this technique’s promise in enhancing methods for protein recognition and separation.

One of the critical issues in the application of MIPCs is the imperative to broaden imprinting techniques to encompass larger biological molecules, notably proteins and living cells. This represents a pivotal avenue for future research, offering the potential to usher in a new era of sensor materials tailored for medical applications. While titanium dioxide is acknowledged for its biocompatibility, its genuine adoption in the realms of biology and clinical testing remains limited. The development of biocompatible TiO_2_ nanomaterials enriched with MIPs is a challenging yet promising pursuit, with far-reaching implications in the medical and biological fields. The selective removal of pharmaceutical contaminants from wastewater continues to be a substantial challenge, despite notable progress, such as the removal of ciprofloxacin using MIL-100-based carriers and MIPs. Further advancements are essential to effectively address a wider spectrum of pharmaceutical pollutants. Furthermore, a fundamental comprehension of the photo-oxidation mechanism holds utmost significance for the advancement of efficient environmental remediation processes. This knowledge serves as the cornerstone for optimizing MIPCs and integrating them seamlessly into wastewater treatment systems. In summary, the field of MIPCs is rapidly evolving, with researchers actively addressing critical challenges related to pollutant removal, biocompatibility, and selective sensing. The continuous development of innovative materials and exploration of novel applications offer great promise for tackling pressing environmental and healthcare challenges in the future.

## 4. Conclusions and Challenges

The current review underscores the growing interest in MIP-enhanced nanomaterials due to their exceptional selectivity. These nanomaterials have been adeptly crafted using advanced technology and surface chemistry. With their remarkable recognition abilities, heightened selectivity, and improved adsorption capacities, they find ideal use in pollutant removal, sensors, and separation. Numerous successful applications of these enhanced nanomaterials are documented in the literature. However, despite these strides, certain challenges remain. These obstacles, such as limited utilization of visible light and applications in biological contexts, hinder the complete potential of MIP-enhanced semiconductors nanomaterials. The key challenges include:

- Enhancing the bond strength and binding capabilities between templates and monomers. Advanced synthesis methods, like controlled radical polymerizations, can optimize the affinity of these materials.
- Ensuring stability and structural integrity of imprinted cavities in MIPs during practical use. Balancing high affinity and cavity resilience is vital, especially in high-porosity polymers that are prone to structural changes affecting binding. Effective imprinting or elution techniques need exploration.
- Mitigating cross-selectivity, where unintended binding to template-similar molecules occurs. The interactions between templates and functional monomers significantly influence the recognition traits of the final matrix. Robust, stable connections with the template should be sought after in monomer selection.
- Despite significant progress in understanding the photodegradation of molecules on imprinted semiconductor nanocomposites, a notable oversight is the lack of differentiation between standard semiconductor nanocomposites and their imprinted versions. While tools like XPS, FTIR, and EPR spectroscopy have been employed to probe the photocatalytic mechanisms of imprinted photocatalysts, there is a pressing need for real-time or continuous monitoring techniques. Such methods are crucial to shed light on how target molecules transition and transform during the photodegradation phase.
- Investigating imprinting techniques to expand target entities from minor molecules to larger biological ones, including proteins and living cells, is vital. While TiO_2_ is considered biologically friendly, its genuine utilization in biological sectors or clinical tests is uncommon. Crafting TiO_2_ nanomaterials that are enhanced with MIPs and are biocompatible is a challenging endeavor. Nevertheless, it holds the promise of ushering in a novel era of sensor materials suited for medical uses.

This review offers thorough instructions for creating typical MIPCs along with insights into new prospects for unique applications involving selective photocatalysis through molecular imprinting in the development of novel MIPCs. As molecular imprinting and materials science advance, it is evident that a growing number of MIP-based photocatalysts possessing exceptional adsorption capabilities and photocatalytic performance will emerge. These advancements hold the potential to effectively target and eliminate environmental pollutants in the times ahead.

## Figures and Tables

**Table 1:** Synthesis overview of surface imprinted photocatalysts.

Materials	Template	Synthesis Method	Application	Ref.
TiO_2_-Fe_3_O_4_	estrone	liquid-phase deposition	The k_app_ value of the target estrone over MIPs was 0.069 min^−1^, being 363% of that over NIPs (0.019 min^−1^) and 238% of that over P25 (0.029 min^−1^).	[12]
TiO_2_ nanotubes	tetracycline hydrochloride (TC)	liquid-phase deposition	The rate constant for TC photodegradation by MIP-TiO_2_ was about 1.9 times higher than that for the TiO_2_ nanotubes. MIP-TiO_2_ photocatalyst gives the kinetics *k* value of degradation of TC about 0.218 h^−1^	[19]
TiO_2_	2,4-D IMID	sol–gel	TiO_2_ MI/2,4D, photodegradation of template about ~47% TiO_2_ MI/IMID, photodegradation of template about ~35%	[36]
La-doped mesoporous titania films	bis-4-nitro-phenyl-phosphate	sol–gel	The reaction catalyzed by the MIF shows a constant rate 4.2171 × 10^−10^ M s^−1^, which is 27% faster than the background hydrolysis and 8% faster than the corresponding NIF.	[38]
TiO_2_/SiO_2_	4-nitrophenol	hydrothermal	The removal efficiency was about 90% at pH 4.	[39]
TiO_2_/WO_3_	2NP	sol–gel	2NP-TiO_2_/WO_3_ *k* constant rate 0.00372 min^−1^ TiO_2_/WO_3_ *k* constant rate 0.0000293 min^−1^	[40]
N-F co-doped TiO_2_	2NP 4NP	ethanol–water solvothermal	2NP-MIP-TiO_2_ *k* constant rate 0.05233 min^−1^ NIP-TiO_2_ *k* constant rate 0.01962 min^−1^	[41]
TiO_2_	2,4-dichlorophenoxyacetic acid	chemical precipitation	When the light is switched on, degradation rate of 2,4D was obtained with the MI TiO_2_/2,4D sample which, after 240 min, degrades ≈ 75% of initial concentration of the herbicide, whereas the bare TiO_2_ degrades ≈ 45% of the pesticide.	[42]
mesoporous TiO_2_	atrazine	evaporation induced self-assembly method (EISA)	Selective photoelectrochemical oxidation of atrazine in complex polluted water samples was successfully achieved on MI-meso-TiO_2_ with removal rate of 91.7%.	[43]
quartz crystal molecularly imprinted TiO_2_	atrazine	sol–gel	The degradation rate constants (K) estimated from the in situ frequency measurement is about 0.62 × 10^−3^s^−1^.	[44]
TiO_2_ nanotubes	9-AnCOOH anthracene-9-carboxylic acid	sol–gel	Thin MIF-TiO_2_ NT 100% Thick MIF-TiO_2_ NT 70%	[45]
TiO_2_	salicylic acid	molecular imprinting technology	Oxidation potentials of salicylic acid: 917 4-methylsalicylic acid: 887	[46]
quartz crystal coated with gold and TiO_2_	4-(4-propyloxyphenylazo)benzoic acid C_3_AzoCO_2_H	sol–gel	The removal efficiency was in the range of 80–90%.	[40]
TiO_2_	ethyl *p*-hydroxybenzoate	sol–gel	The MIP-TiO_2_ degradation rate of ethyl *p*-hydroxybenzoate in 2 h was 81%.	[48]
TiO_2_	salicylic acid	(LPD)	When the concentration of SA is 25 mg L^−1^, the rate constant of SA decomposed over MIF is 0.01189 min^−1^.	[49]
TiO_2_-SiO_2_	RhB	sol–gel	The maximum removal RhB 36.9% was obtained with the EA1TiRhB system (acid catalyzed route with HCl).	[51]
TiO_2_	RhB	sol–gel	The highest obtained adsorption was 28.81 (%) ± 1.22. The highest rate of degradation was 50.72 (%) ± 1.89.	[52]
TiO_2_ microspheres	bilirubin	sol–gel	The rate of photodegradation under UV irradiation: bilirubin (0.0081–0.0118 min^−1^); protoporphyrin (0.0037–0.0051 min^−1^).	[53]
Si doped TiO_2_	catalytic oxytetracycline (OTC)	liquid-phase deposition	The maximum photocatalytic degradation of oxytetracycline wastewater was 80.79% in 120 min.	[54]
Ag/Zn/TiO_2_	ethyl *p*-hydroxybenzoate	sol–gel	The photocatalytic degradation of ethyl *p*-hydroxybenzoate was 99% for 2 h.	[55]
Ag-TiO_2_	ethylparaben	sol–gel	The photocatalytic degradation of ethylparaben was 93.4% in 2 h.	[56]
S–TiO_2_	ethylparaben	sol–gel	The S-EP-TiO_2_ degradation efficiency of methyl orange was 98.58% and of methylene blue 99.81% within 120 min.	[57]
TiO_2_	ethylparaben	modified stepwise acidification	EP photo-degradation efficiency of 87.32%, 89.82% irradiation under UV for 70 min and visible light for 40 h, respectively.	[58]
Fe_3_O_4_/SiO_2_/ hydroxyapatite	simazine	solvothermal	The photodegradation remained as high as 97.2% after eight cycles.	[60]
Pr- TiO_2_	2-sec-butyl-4,6-dinitrophenol (DNBP)	solvothermal	MIP-TMCs and Pr-MIP-TMCs could achieve at least 90% DNBP removal for 300 min of irradiation, while pristine TMCs only achieved about 40% DNBP removal for the same irradiation time.	[61]
TiO_2_-Al_2_O_3_	diethyl phthalate (DEP)	sol–gel	The constant rate for the photodegradation of DEP was 53.0 k_DEP_/10^−3^ min^−1^.	[62]
SiO_2_ –TiO_2_	2,4-dichlorophenol (DCP)	sol–gel method	The degradation efficiency of 2,4-DCP in a single Cu^2+^-doped 2,4-DCP imprinted TiO_2_-SiO_2_ system after 2 h of irradiation was approximately 80%.	[63]
ZnO	acetaminophen	co-precipitation	The degradation efficiency reached 100% for paracetamol (5 × 10^−5^ M) after 3 h. The kinetic constant for the photocatalytic degradation of paracetamol was approximately 1.32 × 10^−2^ min^−1^.	[64]
ZnO doping with Al^3+^ ions	glycerol	sol–gel	The maximum adsorption of glycerol (ca. 9%) is reached at 60 min from the beginning of the adsorption process. At the end of 1 h reaction, the molar fraction of degradation product was 4.5% and the rest was still unconverted glycerol.	[65]
TiO_2_@CNTs	microcystin-LR (MC-LR) a	sol–gel	The detection limit was calculated to be 0.4 pM.	[67]

**Table 2:** Synthesis overview of molecularly imprinted polymers on photocatalysts.

Materials	Template	Synthesis Method	Monomer/Cross-Linker	Application	Ref.
TiO_2_ nanotube arrays	perfluorooctanoic acid	precipitation polymerization	2,2′-azobis (2-methylpropionitrile), ethylene glycol dimethacrylate, 3-methacryloxypropyl trimethoxysilane (MPTS), and 3-aminopropyltriethoxylsilane (APTS), acrylamide	The amount of PFOA adsorbed by the MIP-TiO_2_ NTs was as high as 0.8125 μg/cm^2^. PFOA decomposition and defluorination by the MIP-TiO_2_ NTs reached 84% and 30.2% after 8 h reaction, respectively. The MIP-TiO_2_ NTs could also selectively and rapidly remove PFOA from a secondary effluent, exhibiting a decomposition of 81.1%, almost as high as that observed in pure water.	[27]
NH_2_-MIL-53(Fe) (MOF)	sulfamethoxazole	solvothermal	acrylic acid, methacrylic acid, methyl methacrylate, divinylbenzene, azobisisobutyronitrile	Removal rate of 38.04 mg/g	[66]
TiO_2_ nanotube arrays	perfluorooctanesulfonate	imprinting polymerization	2,2-azobis (2- methylpropionitrile), 3-aminopropyltriethoxylsilane, 3-methacryloxypropyl trimethoxysilane, acrylamide	The limit of detection (LOD)of PFOS (S/N = 3) was calculated to be 86 ng mL^−1^.	[77]
MIL-100 with Fe_3_O_4_	ciprofloxacin	liquid-phase deposition	(3–aminopropyl) triethoxysilanen humic acid	Selectivity _(_α_(QMIP/QNIP))_ = 3.54 Uptake capacity calculated by the Langmuir equation (273.65 mg/g)	[78]
PEDOT/CdS	danofloxacin mesylate	microwave	poly-3,4-ethylenedioxythiophene	Degradation rate of around 84.84%	[79]
POPD-CoFe_2_O_4_	Cu^2+^ ions	microwave	polyo-phenylenediamine, ethyleneglycoldimethacrylate, azobisisobutyronitrile, P123	The highest reduction rate of Cu^2+^ was 45.98%.	[80]
Fe_2_O_3_/chitosan	norfloxacin	microemulsion	chitosan, glutaraldehyde	The system follows the pseudo-first-order kinetic with a k_obs_ value of 0.0012 min^−1^.	[81]
Fe(II)-MOFs@MIP	dibutyl phthalate (DBP)	microemulsion	MAA, AIBN, EGDMA	The recognition level was 169.25 μg × g^−1^. Degradation of DBP (0.071 min^−1^) The selectivity coefficient towards DBP was 7.28 for adsorption and 4.46 for photocatalytic degradation.	[82]
TiO_2_-Pep-poly-L-lysine	pepsin	liquid-phase deposition	poly-L-lysine	The binding constant of pepsin was approximately 7.3 × 10^5^ M^−1^.	[84]
MIPsRhB–PPy/TiO_2_	RhB	in situ polymerization	polypyrrole (PPy)	The rate constant (k) for the photodegradation of RhB over MIP_RhB_-PPy/TiO_2_ is 0.0158 min^–1^.	[97]
MIPs/Co-TiO_2_ nanocomposites.	RhB	solvothermal	p-phenylenediamine, APS ammonium persulfate	The *k* value for the photodegradation of RhB over MIP/Co-TiO_2_ was 0.03606 min^−1^, being 215.7% of RhB over NIP/Co-TiO_2_ nanocomposites (0.01672 min^−1^) and 337.3% of RhB over Co-TiO_2_ nanoparticles (0.01069 min^−1^).	[98]
S-MIP-TiO_2_ NTs	17-β-estradiol	precipitation polymerization	methacrylic acid, trimethylolpropane trimethacrylate, initiator 4,40-azobis(4-cyanovaleric acid)	The removal efficiency was slightly reduced after each use and finally stable at about 84% after six cycles.	[99]
Cu_2_O-doped TiO_2_	diclofenac	precipitation polymerization	MAA, acrylamide	The highest obtained selectivity parameters for adsorption stage (dark-stage) after 1 h was 70,07%. The highest obtained selectivity parameters for photodegradation stage (UV-light) after 2 h was 45,72%.	[100]
TiO_2_ nanotubes	2,4-D	electropolymerization	pyrrole	The detection limit was calculated to be 10 nM (2.2 ng/mL) (S/N = 3).	[101]
TiO_2_ nanotubes	chlorpyrifos	potentiostatic electrodeposition	o-phenylenediamine (o-PD) monomer	The detection limit was calculated to be 0.96 nmol L^−1^.	[102]
B-TiO_2_ NRs	organophosphate pesticide chlorpyrifos CPF	hydrothermal	p-aminothiophenol (functional monomer)	The detection limit was calculated to be 7.4 pg·mL^−1^.	[103]
TiO_2_	propazine (Pro)	precipitation polymerization	ethyleneglycol dimethacrylate, methacrylic acid, 2,2′-azobis (isobutyronitrile)	The adsorption amount of MIP (6.8076 mg g^−1^) when the propazine concentration was 11 mg L^−1^.	[104]
Fe_3_O_4_@TiO_2_@SiO_2_-IIP	Co(II) ions	surface imprinting technique combined with sol–gel process	3-(2-aminoethylamino) propyltrimethoxysilane, tetraethyl orthosilicate	Fe_3_O_4_@TiO_2_@SiO_2_-IIP showed an adsorption capacity for Co(II) of 35.21 mg g^−1^.	[105]
TiO_2_@Lyz	lysozyme (Lyz)	free radical polymerization method	acrylamide/methylene bisacrylamide system hydroxyethyl acrylate/poly(ethylene glycol) dimethacrylate system	The best conditions resulted in an imprinting value of 4.40 and a peak adsorption limit of 120 mg g^−1^ for the TiO_2_@Lyz-MIPs, which was considerably higher than non-imprinted alternatives.	[106]

## Data Availability

No new data were created or analyzed in this study. Data sharing is not applicable to this article.

## List of Abbreviations

MIPCs	molecularly imprinted photocatalysts
AOPs	advanced oxidation processes
EPs	environmental pollutant
MIT	molecular imprinting technology
MIPs	molecularly imprinted polymers
2,4-D	herbicide 2,4-dichlorophenoxyacetic acid
IMID	imidacloprid (1-(6-chloro-3-pyridinylmethyl)- nitro-2-imidazolidinimine
9-AnCOOH	anthracene-9-carboxylic acid
EP	ethyl *p*-hydroxybenzoate
MI	molecularly imprinted
RhB	rhodamine B
TBOT	titanium(IV) butoxide
TTIP	titanium(IV) isopropoxide
TEOS	tetraethyl orthosilicate
OTC	catalytic oxytetracycline
MIF	molecularly imprinted film
DEP	diethyl phthalate
SMX	sulfamethoxazole
PEDOT	poly-3,4-ethylenedioxythiophene
3D	three-dimensional
POPD	polyo-phenylenediamine
NOR	norfloxacin
NIPs	non-imprinted polymers
DBP	dibutyl phthalate
MAA	methacrylic acid
PPy	polypyrrole
APTS	3-aminopropyltriethoxylsilane
MPTS	3-methacryloxypropyl trimethoxysilane
AIBN	azobisisobutyronitrile
EGMA	ethyleneglycoldimethacrylate
TC	tetracycline hydrochloride
LPD	liquid-phase deposition
OPDA	oxylipin 12-oxo-phytodienoic acid
NTs	nanotubes
DIC	diclofenac
PFCs	perfluorinated chemicals
PFOA	perfluorooctanoic acid
PFOS	perfluorooctanesulfonate
2NP	2-nitrophenol
4NP	4-nitrophenol
PEC	photochemistry
MOF	metal organic frameworks
CPF	organophosphate pesticide chlorpyrifos
Lyz	lysozyme
PDA	polydopamine
EISA	evaporation-induced self-assembly method
AA	acrylic acid
MMA	methyl methacrylate
DVB	divinylbenzene
t-Boc	t-butoxycarbonyl
CAR	carbazole
DPP	diketopyrrolopyrrole
